# Identification and characterization of long noncoding RNAs and mRNAs expression profiles related to postnatal liver maturation of breeder roosters using Ribo-zero RNA sequencing

**DOI:** 10.1186/s12864-018-4891-7

**Published:** 2018-06-27

**Authors:** Shengru Wu, Yanli Liu, Wei Guo, Xi Cheng, Xiaochun Ren, Si Chen, Xueyuan Li, Yongle Duan, Qingzhu Sun, Xiaojun Yang

**Affiliations:** 1 College of Animal Science and Technology, Northwest A&F University, Yangling, 712100 China; 2 Dazhou Institute of Agricultural Sciences, Dazhou, 635000 Sichuan China

**Keywords:** Postnatal liver maturation, LncRNAs, Transcriptome, Chicken, Differential expression

## Abstract

**Background:**

The liver is mainly hematopoietic in the embryo, and converts into a major metabolic organ in the adult. Therefore, it is intensively remodeled after birth to adapt and perform adult functions. Long non-coding RNAs (lncRNAs) are involved in organ development and cell differentiation, likely they have potential roles in regulating postnatal liver development. Herein, in order to understand the roles of lncRNAs in postnatal liver maturation, we analyzed the lncRNAs and mRNAs expression profiles in immature and mature livers from one-day-old and adult (40 weeks of age) breeder roosters by Ribo-Zero RNA-Sequencing.

**Results:**

Around 21,939 protein-coding genes and 2220 predicted lncRNAs were expressed in livers of breeder roosters. Compared to protein-coding genes, the identified chicken lncRNAs shared fewer exons, shorter transcript length, and significantly lower expression levels. Notably, in comparison between the livers of newborn and adult breeder roosters, a total of 1570 mRNAs and 214 lncRNAs were differentially expressed with the criteria of log_2_fold change > 1 or < − 1 and *P* values < 0.05, which were validated by qPCR using randomly selected five mRNAs and five lncRNAs. Further GO and KEGG analyses have revealed that the differentially expressed mRNAs were involved in the hepatic metabolic and immune functional changes, as well as some biological processes and pathways including cell proliferation, apoptotic and cell cycle that are implicated in the development of liver. We also investigated the cis- and trans- regulatory effects of differentially expressed lncRNAs on its target genes. GO and KEGG analyses indicated that these lncRNAs had their neighbor protein coding genes and trans-regulated genes associated with adapting of adult hepatic functions, as well as some pathways involved in liver development, such as cell cycle pathway, Notch signaling pathway, Hedgehog signaling pathway, and Wnt signaling pathway.

**Conclusions:**

This study provides a catalog of mRNAs and lncRNAs related to postnatal liver maturation of chicken, and will contribute to a fuller understanding of biological processes or signaling pathways involved in significant functional transition during postnatal liver development that differentially expressed genes and lncRNAs could take part in.

**Electronic supplementary material:**

The online version of this article (10.1186/s12864-018-4891-7) contains supplementary material, which is available to authorized users.

## Background

Liver is the most important metabolic organ exhibiting both endocrine and exocrine properties [1]. However, most of the liver functions are not mature at birth and many changes are extensively remodeled during postnatal liver development to rapidly adapt and perform adult functions [2]. Functional adaption during postnatal liver development is relied on finely programmed alteration of gene expression [3]. Cui et al. [4] and Peng et al. [5] reported that the Cytochrome P450 gene isoforms and their alternative transcripts were closely related to postnatal liver development of mouse. Some other important genes, such as yes-associated protein, aryl, estrogen, and hydroxysteroid sulfotransferases, also could regulate the process of postnatal liver development [6, 7]. Using recently developed RNA sequencing techniques, several genes have been found to involve in postnatal liver development of mouse [8]. However, the differentially expressed genes in chicken liver, which involved in postnatal liver development process and the adaption of mature hepatic metabolic and immune functions, have not been reported before.

It is well-known that protein-coding genes account for only approximately 1.5% of the genome, meaning that most of transcripts have little translation potential [9–11], and these non-coding RNAs could play crucial roles in regulating target gene expressions [11, 12]. As the most important non-protein coding transcripts longer than 200 base pairs, long noncoding RNAs (LncRNAs) are extensively expressed in several species of animals [8, 13–15]. Studies on lncRNAs have been shown to control several levels of the gene expression program, including DNA methylation [16], mRNA expression and degradation [17], and the effective concentration of miRNAs [18], by acting as signal, decoys, guides, scaffolds [11], or competing endogenous RNA [18]. These results have highlighted the regulatory roles of lncRNAs in regulating epigenetic modification and gene expression. However, compared with the extensive characterization of DNA methylation and alternative pre-mRNA splicing related to fetal-to-adult liver maturation [19, 20], the roles of lncRNAs in regulating postnatal liver maturation need to pay more attentions.

RNA-seq technology has rapidly developed to enable discovery and analysis of non-coding RNA, and differential methods have been developed to identify novel lncRNAs using RNA-seq data. With oligo (dT) selection of poly (A)^+^ mRNA, recent studies in mice have focused on the changes of lncRNAs or mRNAs related to the regulation of postnatal live development and functional changes [8, 21, 22]. However, not all of the lncRNAs contains 3’polyadenylation [23]. Thus it could ignore some lncRNAs information by using poly (A) selection methods to prepare cDNA library. Compared with poly (A) selection Sequencing, Ribo-Zero RNA Sequencing, which provided equivalent rRNA removal efficiency and coverage uniformity but exhibited a highly technical reproducibility, can help study poly (A)^−^ mRNA, immature transcripts, and the lncRNAs [24, 25]. In contrast to the small RNAs, which are highly conserved and involved in transcriptional and posttranscriptional gene silencing through specific base pairing with their targets, lncRNAs are poorly conserved and regulate gene expression by diverse mechanisms, suggesting primarily lineage-specific functions [26].

Hence, we hypothesized that postnatal liver maturation of chicken were related to their differential lncRNA expression profiles. Herein, we investigated the expression profiles of lncRNAs and mRNAs related to postnatal liver maturation of chicken by Ribo-Zero RNA-Seq [27] by using 6 livers transcriptome libraries from arbor acres roosters, which is one of the most used broiler breeder roosters. Taken together, these expression profiles could especially clarify the changes of mRNAs related to postnatal liver maturation and the roles of lncRNAs in postnatal liver development, metabolism, and other liver functions.

## Results

### RNA sequencing and identification of mRNA and lncRNAs in chicken liver

**Fig. 1:**
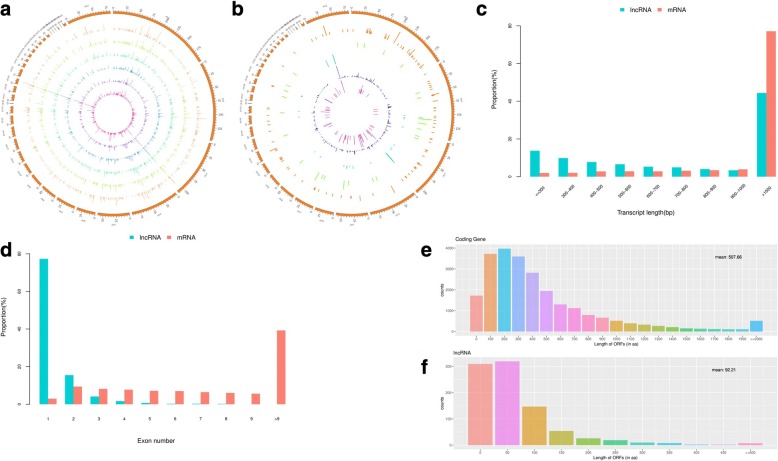
Characteristics of lncRNAs in the livers of chickens (*Gallus gallus*). **a**. the expression level of lncRNAs (log_10_FPKM) along the *Gallus gallus* chromosomes. It comprises six concentric rings, and each corresponds to a different sample. They are mature livers (M1, M2, and M3) and immature livers (IM1, IM2, and IM3) from outer to inner, respectively. **b**. Distribution of different types of lncRNAs. The antisense, intron, intronic, sense, and intergenic lncRNAs are represented by different concentric rings from inner to outer, according to the loci of lncRNAs in the genome. **c**. Length distribution of lncRNAs and mRNAs. **d** Exon number distribution of protein coding transcripts and lncRNAs. **e** and **f**. ORFs length distribution of coding transcripts and lncRNAs, and the average value were shown in these two figures

To systematically identify mRNAs and lncRNAs expressed in the mature and immature livers of breeder roosters, we generated six RNA expression profiles of liver tissues with an average of 87 million 150 bp paired end raw reads. After initial processing, the average of 58 million valid reads were obtained from each sequencing library (Table 1). By using TopHat [28], nearly 86% of the reads were mapped to the *Gallus gallus* reference genome (Galgal5). The mapped sequences in each library were assembled and annotated using the StringTie [29]. In the present study, a total of 20,158, 19,726, 19,751, 19,351, 19,543, and 19,687 unique genes from the six libraries were respectively identified (Table 1 and Additional file 1). According to length and coding potentials (see methods), we identified 1918, 1927, 1932, 2019, 2042, and 2022 unique lncRNAs from those six libraries (Table 1 and Additional file 2). These 2220 unique lncRNAs were distributed across the chromosomes in *Gallus gallus* related to the length of the chromosomes (Fig. 1a). According to the locations of lncRNAs in the genome, 80 antisense, 49 sense, 1922 intergenic, 32 intronic, and 137 intron lncRNAs were identified among our identified lncRNAs (Fig. 1b). Moreover, the sequence information of all identified lncRNAs were listed in Additional file 3.

**Table 1:** Statistical data of the RNA-Sequencing reads for six samples

	Mature liver			Immature liver		
	M1	M2	M3	IM1	IM2	IM3
Q20 (%)	99.53	99.55	99.51	99.56	99.47	99.52
Q30 (%)	95.48	95.49	94.78	95.66	94.67	94.89
GC content (%)	51	52.5	50	50	51	50
Raw reads	85,224,338	90,000,000	76,894,960	90,000,000	89,618,576	90,000,000
Valid reads	73,446,942	78,312,446	66,231,654	64,371,012	56,267,806	66,562,834
Mapped reads	67,733,671 (92.22%)	69,790,614 (89.12%)	58,100,222 (87.72%)	55,492,843 (86.21%)	44,247,356 (78.64%)	53,016,014 (79.65%)
Unique genes	20,158	19,726	19,751	19,351	19,543	19,687
Unique lncRNAs	1918	1927	1932	2019	2042	2022

In the present study, the average length of lncRNAs was 1718 bp compared to more than 2936 bp for protein-coding genes, which indicated that lncRNAs were shorter than protein coding transcripts (Fig. 1c). Genes of lncRNAs tend to contain fewer exon: lncRNAs identified in our study had only 1.35 exons per transcript on average while protein-coding genes had averaged 10.15 exons (Fig. 1d). Furthermore, the lncRNAs in chicken livers tended to be shorter in length of open reading frame than protein coding genes (Fig. 1e and f). Overall, lncRNAs identified in our study were characterized with fewer exons, shorter transcript length, and significantly lower expression levels, compared to protein-coding genes, which is consistent with previous studies in other species as well as in chickens [30–32].

### Developmental changes of protein-coding genes and lncRNAs in postnatal liver development of chicken

**Fig. 2:**
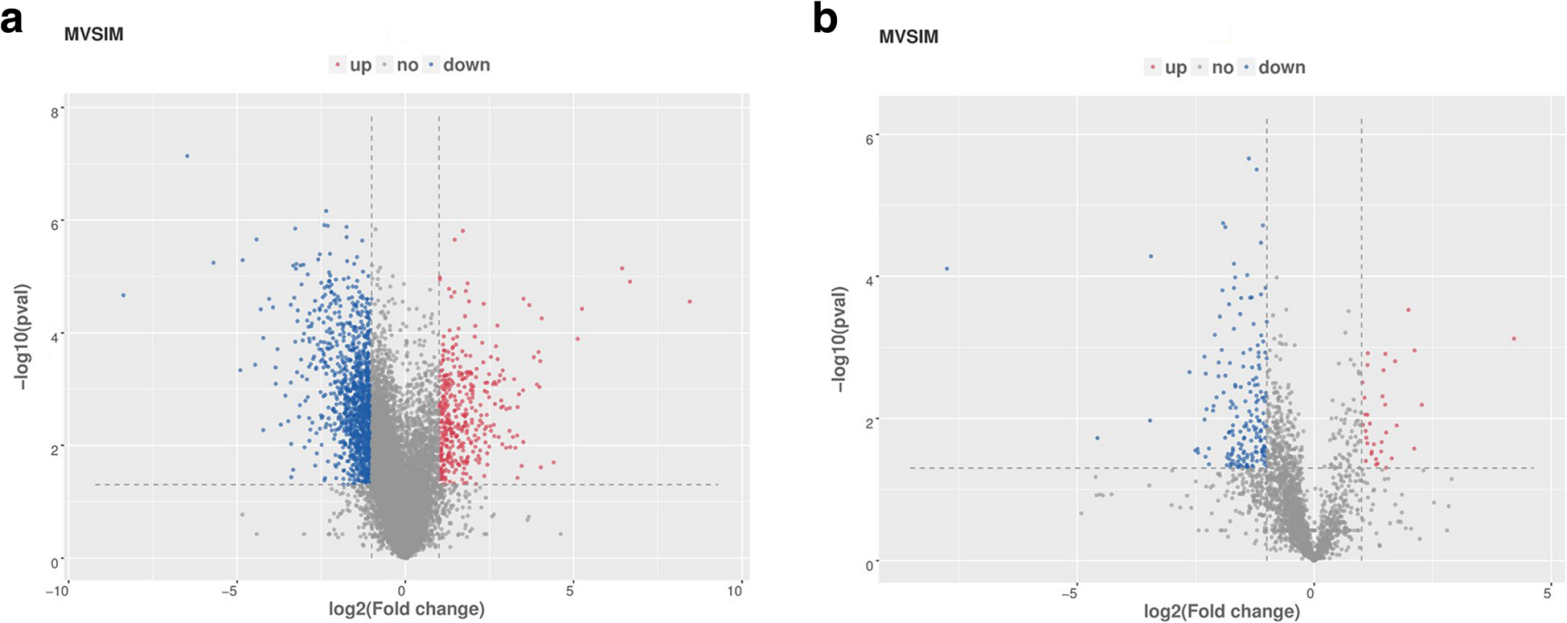
The differential expression of chicken mRNAs and lncRNAs between immature livers and mature livers are shown. **a** differential expression of mRNAs. the left blue points represent significantly decreased mRNAs in immature livers, gray points represent mRNAs without significantly changes, the right red points represent significantly increased mRNAs in immature livers. **b** differential expression of lnRNAs. the left blue points represent significantly decreased lncRNAs in immature livers, gray points represent lncRNAs without significantly changes, the right red points represent significantly increased lncRNAs in immature livers

We gathered 1570 differentially expressed mRNAs between immature and mature livers meeting the criteria of *P* < 0.05 and log_2_foldchange > 1 or < − 1. Of these, 381 genes were up-regulated and 1189 mRNAs were down-regulated in the mature livers relative to the immature livers in breeder roosters (Fig. 2a and Additional file 4). In addition, 214 differentially expressed lncRNAs between immature and mature livers meeting the criteria of *P* < 0.05 and log_2_foldchange > 1 or < − 1 were obtained. Compared with the immature livers, 34 up-regulated lncRNAs and 180 down-regulated lncRNAs were obtained in mature livers (Fig. 2b and Additional file 5).

**Fig. 3:**
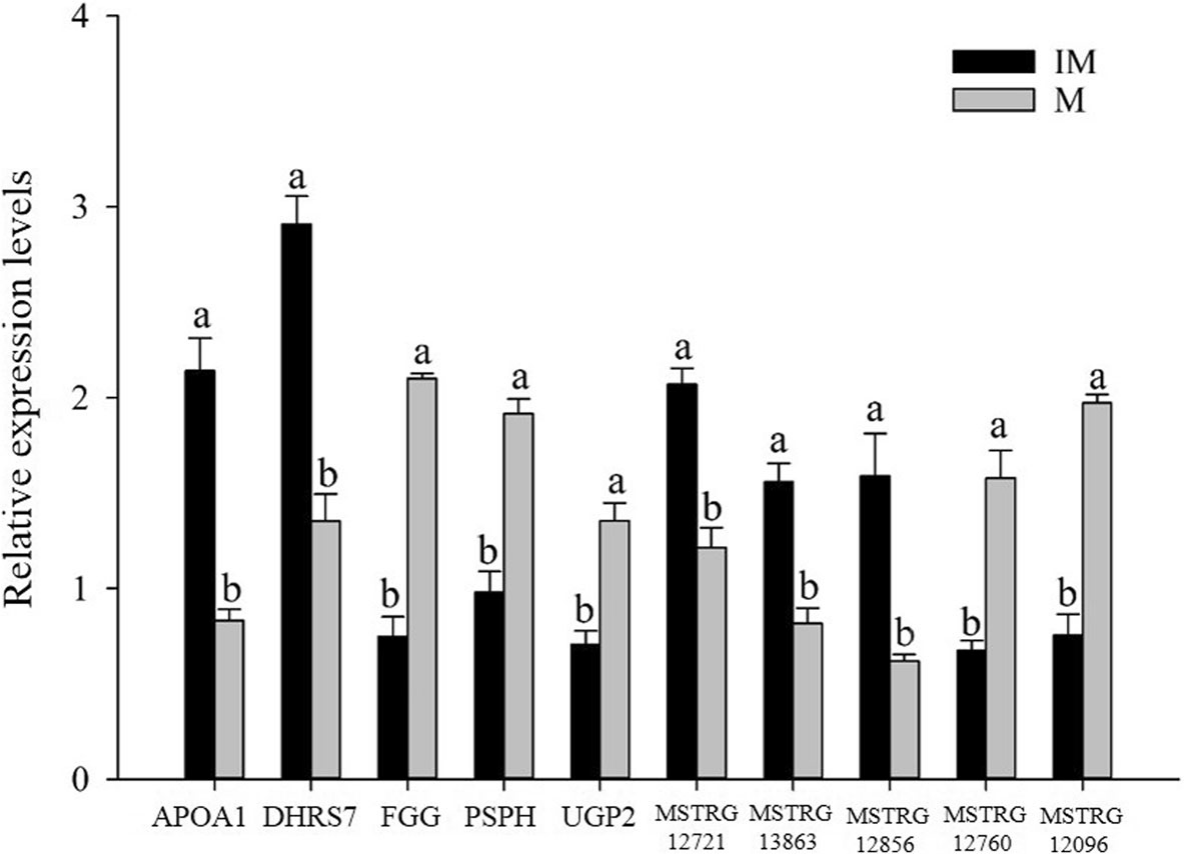
Five differentially expressed mRNAs and five differentially expressed lncRNAs, which were validated by reverse-transcription quantitative polymerase chain reaction. 1. IM represent the immature livers from one-day-old chickens, M represent the mature livers from adult chickens; 2. β-actin was used as an internal control gene for normalization in our experiments. The data were presented as means ± SE (for young chicks: *n* = 20; for adult chickens: *n* = 5). Upper letters (a, b) on bars denote significantly different expression levels in the same mRNAs or lncRNAs (*P* < 0.05)

The expression levels of ten randomly selected lncRNAs and mRNAs were determined by quantitative real-time PCR. The results confirmed that these lncRNAs and mRNAs were expressed at both mature and immature livers (Fig. 3) and showed differential expression at different stages. In addition, the qRT-PCR confirmed that the expression patterns of these lncRNAs and mRNAs were consistent with their expression levels calculated from the RNA-seq data (Fig. 3).

### Enrichment analysis of differentially expressed mRNAs

GO enrichments [33] of the differentially expressed mRNAs were categorized into 519 functional annotations that met the criteria of *P* < 0.05 (Additional file 6). Results of the biological process analysis revealed that these differentially expressed mRNAs could mainly regulate the biological process relative to metabolism, immune response, oxidation-reduction process, as well as some process related to the growth and functional maturation of liver, including the cell division, proliferation, apoptotic, and cell cycle. Moreover, analyses based on cellular components and molecular functions showed that differentially expressed mRNAs were also involved in the aforementioned process relative to the growth and functional maturation of liver. Further KEGG pathway [34] analyses revealed that these mRNAs were mostly involved in several pathways affecting the metabolism of livers, including the amino acid metabolism, glycometabolism, and lipid metabolism, as being listed in Table 2. Furthermore, pathways relative to the growth of liver, such as cell cycle pathway, were also found to take part in the postnatal liver maturation in the present study.

**Table 2:** The significantly enriched KEGG pathways (with *P* < 0.05) based on the differentially expressed mRNAs

Pathway Id	pathway description	S gene number	TS gene number	B gene number	TB gene number	P value
ko00190	Oxidative phosphorylation	34	579	88	3735	0.00
ko00280	Valine, leucine and isoleucine degradation	19	579	42	3735	0.00
ko01200	Carbon metabolism	30	579	88	3735	0.00
ko03030	DNA replication	13	579	27	3735	0.00
ko00640	Propanoate metabolism	13	579	28	3735	0.00
ko03320	PPAR signaling pathway	19	579	51	3735	0.00
ko00630	Glyoxylate and dicarboxylate metabolism	12	579	26	3735	0.00
ko01230	Biosynthesis of amino acids	19	579	53	3735	0.00
ko00620	Pyruvate metabolism	13	579	31	3735	0.00
ko00071	Fatty acid degradation	12	579	29	3735	0.00
ko04216	Ferroptosis	12	579	30	3735	0.00
ko00260	Glycine, serine and threonine metabolism	12	579	33	3735	0.00
ko00020	Citrate cycle (TCA cycle)	10	579	25	3735	0.00
ko01212	Fatty acid metabolism	13	579	39	3735	0.00
ko00650	Butanoate metabolism	9	579	23	3735	0.01
ko00380	Tryptophan metabolism	11	579	34	3735	0.01
ko00072	Synthesis and degradation of ketone bodies	5	579	10	3735	0.01
ko00310	Lysine degradation	12	579	39	3735	0.01
ko04110	Cell cycle	24	579	99	3735	0.01
ko00240	Pyrimidine metabolism	20	579	79	3735	0.02
ko04146	Peroxisome	17	579	65	3735	0.02
ko03430	Mismatch repair	7	579	19	3735	0.02
ko04914	Progesterone-mediated oocyte maturation	17	579	67	3735	0.02
ko04145	Phagosome	25	579	109	3735	0.02
ko00220	Arginine biosynthesis	6	579	16	3735	0.03
ko01210	2-Oxocarboxylic acid metabolism	5	579	12	3735	0.03
ko00480	Glutathione metabolism	10	579	34	3735	0.03
ko00330	Arginine and proline metabolism	10	579	34	3735	0.03
ko04217	Necroptosis	22	579	95	3735	0.03
ko03440	Homologous recombination	10	579	36	3735	0.04
ko00983	Drug metabolism - other enzymes	7	579	22	3735	0.04
ko00980	Metabolism of xenobiotics by cytochrome P450	8	579	27	3735	0.05
ko00900	Terpenoid backbone biosynthesis	6	579	18	3735	0.05
ko00010	Glycolysis / Gluconeogenesis	11	579	42	3735	0.05

S gene number: the number of significant differentially expressed mRNAs which match to a KEGG term; TS gene number: the number of significant differentially expressed mRNAs which have KEGG annotations; B gene number: the number of detected mRNAs which match to a KEGG term; TB gene number: the number of all detected mRNAs which have KEGG annotations

### Cis-regulatory roles and trans-regulatory roles of differentially expressed lncRNAs in postnatal liver maturation of chicken

To investigate the possible functions of the lncRNAs, we predicted the potential targets of lncRNAs in cis-regulatory and trans- regulatory relationships. In the present study, 4 and 214 differentially expressed lncRNAs could respectively cis- and trans-regulate 4 and 1394 differentially expressed mRNAs in the present study (Additional file 7). GO analysis based on these cis- and trans- regulated targets was performed and 174 significant enriched (*P* < 0.05) GO terms were obtained (Additional file 8). As the main function of mature liver after birth, several biological processes and molecular function related to metabolic and immune process have also been detected. Results also revealed that these differentially expressed lncRNAs could cis- or trans- regulate targets involved in liver development, including liver development process, cell adhesion, cell proliferation, and apoptotic processes. KEGG pathway analyses showed that a total of 153 KEGG pathways were annotated based on the cis- and trans- regulated differentially expressed targets of differentially expressed lncRNAs. Of these, 25 KEGG pathways were annotated with *P* < 0.05 (Table 3), most of which were related to the regulation of mature hepatic metabolic and immune functions. Moreover, some pathways, which have been proved to regulate liver development, have also been detected in the KEGG analyses of differentially expressed lncRNAs, such as the cell cycle, Notch signaling pathway, Wnt signaling pathway, and Hedgehog signaling pathway. These analyses indicated that the differentially expressed lncRNAs and their annotated pathways should play cis- or trans- regulated roles in postnatal liver maturation. Moreover, the interaction between lncRNAs and their cis- or trans- regulated target genes which involved in different GO terms and KEGG pathways were also respectively listed in Additional file 7.

**Table 3:** The KEGG pathway analysis (with *P* < 0.05) based on the cis- and trans-regulated differentially expressed targets of differentially expressed lncRNAs

Pathway Id	pathway description	S gene number	TS gene number	B gene number	TB gene number	*P* value
ko00190	Oxidative phosphorylation	74	531	76	689	0.000
ko04514	Cell adhesion molecules (CAMs)	34	531	35	689	0.001
ko00280	Valine, leucine and isoleucine degradation	32	531	33	689	0.002
ko00240	Pyrimidine metabolism	38	531	40	689	0.002
ko00620	Pyruvate metabolism	30	531	31	689	0.003
ko04260	Cardiac muscle contraction	22	531	22	689	0.003
ko05168	Herpes simplex infection	42	531	45	689	0.003
ko04210	Apoptosis	40	531	43	689	0.005
ko00270	Cysteine and methionine metabolism	20	531	20	689	0.005
ko00020	Citrate cycle (TCA cycle	20	531	20	689	0.005
ko01200	Carbon metabolism	60	531	67	689	0.005
ko01230	Biosynthesis of amino acids	38	531	41	689	0.007
ko04672	Intestinal immune network for IgA production	18	531	18	689	0.009
ko03010	Ribosome	56	531	63	689	0.011
ko00640	Propanoate metabolism	24	531	25	689	0.011
ko00230	Purine metabolism	45	531	50	689	0.014
ko00970	Aminoacyl-tRNA biosynthesis	16	531	16	689	0.015
ko03320	PPAR signaling pathway	34	531	37	689	0.016
ko04217	Necroptosis	28	531	30	689	0.018
ko01212	Fatty acid metabolism	22	531	23	689	0.018
ko00630	Glyoxylate and dicarboxylate metabolism	22	531	23	689	0.018
ko05164	Influenza A	38	531	42	689	0.020
ko00480	Glutathione metabolism	20	531	21	689	0.029
ko04216	Ferroptosis	12	531	12	689	0.043
ko00983	Drug metabolism - other enzymes	12	531	12	689	0.043

S gene number: the number of significant differentially expressed lncRNAs whose cis-or trans- regulated targets match to a KEGG term; TS gene number: the number of significant differentially expressed lncRNAs whose cis- or trans- regulated targets have KEGG annotations; B gene number: the number of detected lncRNAs whose cis- or trans- regulated targets match to a KEGG term; TB gene number: the number of all detected lncRNAs whose cis- or trans- regulated targets have KEGG annotations

### Integrated analyses of differentially expressed lncRNAs and mRNAs involved in postnatal liver development

Several differentially expressed protein-coding genes involved in liver development, such as SNX, CYP7A1, CYP39A1, HNF4α, and IGF2BP1, could be cis-regulated or trans-regulated (Table 4) by several differentially expressed lncRNAs. Furthermore, we can find that several key genes of some important pathways involved in liver development, including the cell cycle, Notch signaling pathway, Wnt signaling pathway, and Hedgehog signaling pathway, could also be regulated by the lncRNAs in cis-role or trans-role (Additional file 9). Overall, we suspected that these lncRNAs most probably participated in the postnatal liver development, although its underlying mechanisms require additional investigations.

**Table 4:** LncRNAs and its potential target genes that are involved in postnatal liver development

Key protein coding gene	lncRNAs in cis- or trans-roles
SNX	MSTRG.5900.3/MSTRG.12856.1/MSTRG.12870.1/MSTRG.13153.1/MSTRG.13191.1/MSTRG.13327.1/MSTRG.13411.1/MSTRG.13471.1/MSTRG.13567.1/MSTRG.13579.1/MSTRG.13597.1/MSTRG.13748.1/MSTRG.13863.1/MSTRG.14503.1/MSTRG.14734.1/MSTRG.15377.1/MSTRG.15517.1/MSTRG.16154.4/MSTRG.16668.1
CYP7A1	MSTRG.636.1/MSTRG.1039.1/MSTRG.1041.1/MSTRG.1226.1/MSTRG.1392.1/MSTRG.1438.2/MSTRG.7038.1/MSTRG.8045.2/MSTRG.11864.1/MSTRG.12193.1/MSTRG.12936.1/MSTRG.13046.1/MSTRG.13286.1/MSTRG.13438.1/MSTRG.13472.1/MSTRG.13797.1/MSTRG.14192.1/MSTRG.14360.1/MSTRG.14681.1/MSTRG.14723.1/MSTRG.15490.1/MSTRG.15512.1/MSTRG.15585.1/MSTRG.15717.1/MSTRG.15774.1/MSTRG.15789.1/MSTRG.15824.1/MSTRG.16055.1/MSTRG.16119.1/MSTRG.16447.2/MSTRG.16938.1
CYP39A1	MSTRG.1064.1/MSTRG.3562.1/MSTRG.5948.1/MSTRG.12710.1/MSTRG.12721.1/MSTRG.12856.1/MSTRG.12870.1/MSTRG.13006.1/MSTRG.13157.1/MSTRG.13191.1/MSTRG.13267.1/MSTRG.13298.1/MSTRG.13319.1/MSTRG.13353.1/MSTRG.13411.1/MSTRG.13426.1/MSTRG.13471.1/MSTRG.13515.1/MSTRG.13518.1/MSTRG.13567.1/MSTRG.13597.1/MSTRG.13603.1/MSTRG.13692.1/MSTRG.13748.1/MSTRG.13853.1/MSTRG.13863.1/MSTRG.14018.1/MSTRG.14126.1/MSTRG.14408.1/MSTRG.14481.1/MSTRG.14666.1/MSTRG.14727.1/MSTRG.14734.1/MSTRG.14805.1/MSTRG.14829.1/MSTRG.14838.1/MSTRG.14895.1/MSTRG.15517.1/MSTRG.15518.1/MSTRG.15756.1/MSTRG.15797.1/MSTRG.16379.1/MSTRG.16495.1/MSTRG.16668.1/MSTRG.16961.1/MSTRG.16958.27/MSTRG.16941.1
HNF4α	MSTRG.1039.1/MSTRG.1226.1/MSTRG.1438.2/MSTRG.2310.1/MSTRG.4207.1/MSTRG.5597.1/MSTRG.7038.1/MSTRG.8045.2/MSTRG.10533.1/MSTRG.11864.1/MSTRG.12193.1/MSTRG.12709.1/MSTRG.12721.1/MSTRG.12870.1/MSTRG.13046.1/MSTRG.13222.1/MSTRG.13267.1/MSTRG.13319.1/MSTRG.13353.1/MSTRG.13438.1/MSTRG.13518.1/MSTRG.13567.1/MSTRG.13603.1/MSTRG.13824.1/MSTRG.13863.1/MSTRG.13877.1/MSTRG.13942.1/MSTRG.13960.1/MSTRG.14360.1/MSTRG.14684.1/MSTRG.14723.1/MSTRG.14733.1/MSTRG.14734.1/MSTRG.14838.1/MSTRG.14869.1/MSTRG.15511.1/MSTRG.15512.1/MSTRG.15517.1/MSTRG.15518.1/MSTRG.15585.1/MSTRG.15789.1/MSTRG.16055.1/MSTRG.16119.1/MSTRG.16273.2/MSTRG.16347.1/MSTRG.16447.2/MSTRG.16461.3/MSTRG.16495.1/MSTRG.16941.1
IGF2BP1	MSTRG.1039.1/MSTRG.1041.1/MSTRG.1226.1/MSTRG.1392.1/MSTRG.1438.2/MSTRG.2310.1/MSTRG.4207.1/MSTRG.5597.1/MSTRG.8045.2/MSTRG.10533.1/MSTRG.11864.1/MSTRG.12193.1/MSTRG.12709.1/MSTRG.12870.1/MSTRG.13046.1/MSTRG.13222.1/MSTRG.13319.1/MSTRG.13438.1/MSTRG.13567.1/MSTRG.13603.1/MSTRG.13640.1/MSTRG.13824.1/MSTRG.13863.1/MSTRG.13877.1/MSTRG.13942.1/MSTRG.14176.1/MSTRG.14360.1/MSTRG.14723.1/MSTRG.14733.1/MSTRG.14869.1/MSTRG.15104.1/MSTRG.15390.1/MSTRG.15435.1/MSTRG.15511.1/MSTRG.15512.1/MSTRG.15517.1/MSTRG.15518.1/MSTRG.15585.1/MSTRG.15717.1/MSTRG.15789.1/MSTRG.15833.1/MSTRG.16055.1/MSTRG.16119.1/MSTRG.16273.2/MSTRG.16347.1/MSTRG.16461.3/MSTRG.16495.1/MSTRG.16941.1

## Discussion

Breeder roosters and their offspring broilers (*Gallus gallus*) are famous for its high feed conversion efficiency, which means a high efficient metabolic process in chicken liver [35]. There are several serious metabolic diseases occurred during the feeding of breeder roosters, such as fatty liver and ascites syndrome [36], which are induced by incorrect metabolic regulation and could further influence the usability of breeder roosters. It’s important to clarify the postnatal liver maturation process and the hepatic function changes after birth, so that the hepatic metabolic or immune condition of breeder roosters could be better regulated and the breeder roosters could be used maximally. However, compared with abundant researches on liver development of human or mice [8, 21, 22, 37–40], limited researches have focused on the postnatal liver maturation process in chicken. In the present study, we identified the differentially expressed mRNAs and lncRNAs in immature and mature livers of breeder roosters by using RIBO-zero RNA-Sequencing, which is the first report to systematically identify mRNAs and lncRNAs expression profiles during chicken postnatal liver development.

Postnatal liver development from the newborn to the adult stage consists of a series of exquisitely regulated and orchestrated changes in the expression of many genes. In the previous studies, we can easily find that several differentially expressed mRNAs have been found to be involved in liver maturation in other species of animals, such as sorting nexin (SNX) in zebra fish [41], insulin like growth factor 2 mRNA binding protein 1 (IGF2BP1) in human [42], as well as hepatocyte nuclear factor 4-alpha (HNF4α) [43] and cytochrome P450 gene isoforms [4, 5] in mice. These genes could be detected in our identified differentially expressed mRNAs, which indicted that these differentially expressed mRNAs have important roles in regulating livers development of chickens, as being shown in other animal species. Further GO and KEGG analyses indicated that differentially expressed mRNAs regulated many metabolic and immune processes in liver, which are related to normal mature liver functions [1, 44, 45] and were consist with the results of previous studies in mice [8, 21, 22]. Except for the metabolic and immune changes with the maturation of liver, we found that there were several GO terms involved in the oxidation-reduction process and antioxidant ability, which was also the main function of mature liver [46]. Moreover, the majority of genes in the liver cell proliferation process, such as the genes involved in cell cycle, DNA replication, cytokine binding, cell proliferation, and cell division(including cytokinesis, chromosome segregation, and mitotic nuclear division), displayed remarkable changes in expression during chicken liver development, which highlighted the importance of gene products in postnatal liver growth process [47, 48]. Specifically, the cell cycle pathway have been implied in the liver regeneration process [49, 50]. The liver regeneration is the compensatory growth of the liver, which indicated that the growth of liver could also be influenced by the cell cycle pathway. Sadler et al. [51] have reported that the uhrf1 gene, a cell cycle regulator, is required for physiologic liver growth in both embryos and adults in zebrafish. The uhrf1 was also been detect as the differentially expressed genes in the present study. These results again proved that the cell cycle pathway were involved in the postnatal liver growth process. To sum up, GO and KEGG analyses based on differentially expressed mRNAs indicated that livers underwent hypertrophic growth and maturation via large-scale changes in metabolic and immune functions after birth.

LncRNAs are a group of endogenous RNAs involved in developmental and physiological processes [52–54]. We obtained 300 up-regulated lncRNAs and 322 down-regulated lncRNAs in mature livers. These lncRNAs may have specific biological roles in postnatal liver development in chickens. Several recent studies have proved that lncRNAs could play crucial roles in liver development by using the RNA sequencing [8, 55, 56]. Compared with these previous studies, lncRNAs with or without poly (A) tails were obtained using RIBO-zero RNA-Sequencing in the present study. More types of lncRNAs, including those of sense, antisense, intronic and intergenic lncRNAs, were identified, while some previous studies only obtained the information of long intergenic non-coding RNAs. However, limit research proved the roles of single lncRNA in liver development process. Only the lncRNA-LALR1 were proved to enhance hepatocyte proliferation by promoting progression of the cell cycle, and further accelerate mouse hepatocyte growth and cell cycle progression during liver growth [57]. In addition, lncRNA-LALR1 facilitated cyclin D1 expression through activation of Wnt/β-catenin signaling by way of suppression of Axin1 [57]. Another study revealed that a set of lncRNAs highly correlated with expression of cytochrome P450, which alter their expressions during liver development and have critical functions in liver to metabolize xeno−/endo-biotics [58]. .Therefore, the differentially expressed lncRNAs reported in the present study can also be considered as important novel regulators of chicken postnatal liver development process.

On the one hand, most evidence suggests that the expression of lncRNAs can regulate and have high correlations with expression of neighboring mRNAs in animals, microorganism, and plants [42, 59, 60]. On the other hand, many lncRNAs can also function in trans mode to target gene loci distant from where the lncRNAs are transcribed [11, 61]. In this study, the cis- and trans-regulated targets of the differentially expressed lncRNAs were obtained. There were several differentially expressed mRNAs related to liver development, such as SNX [41], IGF2BP1 [42], CYP7A1, CYP39A1 [4, 5], and HNF4α [43], could be regulated by differentially expressed lncRNAs in either cis or trans roles. These findings indicated that lncRNAs could cis- and trans-regulated the protein-coding genes associated with postnatal liver maturation. Further GO and KEGG analyses for trans- and cis-regulatory roles of differentially expressed lncRNAs were performed and found these lncRNAs could regulate the cell proliferation, cell cycle, as well as several liver function, such as metabolism, immunity, and antioxidant, which was consist with the results of functional annotation of differentially mRNA. These results indicated that the hepatic mature function could be regulated by the differentially expressed lncRNAs found in our study. We further found that the differentially expressed lncRNAs could regulate several pathways that have proven to be involved in liver development, such as Notch pathway, Hedgehog pathway, adherens junction pathway, and Wnt signaling pathway. In the previous studies, Zong et al. [62] and Kodama et al. [63] have proven that the Notch pathway were involved in the liver development by regulating biliary differentiation. Tanimizu and Miyajima [64] suggested that Notch signaling could control hepatoblast differentiation by altering the expression of liver-enriched transcription factors. As for hedgehog pathway, it is well known for its mitogenic and morphogenic functions during development [65]. Reactivation of Hedgehog, a signaling pathway that controls hepatic progenitor cell fate and tissue construction, have also been linked to the regulation of adult liver tissue homeostasis, repair, and development [66, 67]. Further research have also proven that the Notch and Hedgehog pathways could interact to control the fate of hepatic key cell types involved in adult liver repair [68]. Moreover, adherens junction pathway and Wnt signaling pathway were been linked to liver development as well [69–71]. Therefore, the differentially expressed lncRNAs involved in these above pathways (Additional file 9) could play important roles in postnatal liver development and were worthy of further research to illuminate their roles in liver development.

## Conclusions

In conclusion, we firstly obtained high-quality mRNA and lncRNA expression profiles in chicken liver based on a RIBO-zero RNA-seq approach and have identified differentially expressed mRNAs and lncRNAs related to postnatal liver maturation of chicken. Moreover, we firstly reported the functional annotation of differentially expressed mRNAs and found that these some genes potentially play an important role in the development of chicken liver and the regulation of the functions of mature livers. Moreover, bioinformatics analysis suggests that some lncRNAs are involved in important biological processes and pathways associated with liver development such as cell cycle pathway, Notch signaling pathway, Hedgehog signaling pathway, and Wnt signaling pathway, and also could play an important role in regulating the gene expression of mature liver functions. Our results not only reveal new information regarding the development of chicken liver but also provide a broad and novel vision for future research at the molecular level in chicken.

## Methods

### Animals and sample collections

All experimental protocols and animals’ managements in the study were approved by the Institutional Animal Care and Use Committee (IACUC) of the Northwest A&F University (Yangling, Shaanxi, China). Five adult healthy Arbor Acres breeder roosters (40 week of age) and Twenty one-day-old healthy Arbor Acres breeder roosters were collected from Experimental Teaching Center of Animal Science of the Northwest A&F University (Yangling, Shaanxi, China). Here, the Arbor Acres breeder roosters in 40 week age were both somatic and sexual matured, which ensured their livers were assuredly matured. All 40-week-old breeder roosters were kept in an environmentally controlled henhouse with double-floor metabolism cages and exposed to a 16 h photoperiod. Water was available ad libitum and food was available according to the feeding standard of Arbor Acres breeder roosters (Aviagen, Alabama, USA).

These randomly selected roosters were fed deprived for 12 h, then euthanized by exsanguination after intravenous (IV) administration of 3% sodium pentobarbital (25 mg/kg; Sigma, USA) and immediately dissected. All efforts were made to minimize animals’ suffering. The whole left side livers were collected into Eppendorf tubes, and frozen immediately in liquid nitrogen. For each liver sample we gathered, the liver was grinded and homogenized using liquid nitrogen, and then all the homogenized liver samples were stored at − 80 °C until be analyzed. The livers of three adult roosters and twelve one-day-old roosters were selected randomly for RNA isolation and next generation sequencing analyses; furthermore, all livers samples from five adult roosters and twenty one-day-old roosters were used to extract total RNA and perform quantitative RT-PCR validation.

### RNA isolation and sequencing

Total RNA from 3 adult roosters’ and 12 one-day-old rooster’ livers for RNA sequencing were extracted using Trizol reagent (Invitrogen, CA, USA) according to the manufacturer’s procedure. Specifically, the DNaseI was used during the RNA isolation process to avoid contamination with genomic DNA. The quantity and purity of total RNA were analyzed by a NanoDrop® ND-1000 spectrophotometer (Thermo Scientific, MA, USA), and integrity of RNA was accessed with Bioanalyzer 2100 and RNA Nano6000 LabChip Kit (Agilent, CA, USA). Only samples that had the OD260/280 > 1.8, OD260/230 > 2.0, and the RNA Integrity Number (RIN) > 7.0 were used for further sequencing (Additional file 10). Four RNA samples of one-day-old chicks’ liver were mixed equally together as a pooled RNA sample according to the purity of total RNA, and the RNA samples of adult chickens’ livers were directly used for library construction. In total, we gathered three RNA samples from three adult roosters and three pooled RNA samples from 12 one-day-old roosters for further library construction.

Approximately 3 μg of total RNA was used to prepare an LncRNA library. According to protocol of Epicentre Ribo-zero™ Gold Kit (Illumine, San Diego, USA), ribosomal RNA was removed and the rRNA-depleted RNA (Poly A^+^ and Poly A^−^ RNA) were collected [24, 25, 42]. Subsequently, high strand-specificity libraries were generated using the rRNA-depleted RNA and a NEBNext Ultra Directional RNA Library Prep Kit for Illumina (NEB, Ipswich, MA, USA) following the manufacturer’s recommendations. Briefly, the rRNA-depleted RNA was fragmented using divalent cations under elevated temperature in NEBNext. First-strand cDNA was synthesized using random hexamer primers and M-MuLV reverse transcriptase (RNase H−). Subsequently, second-strand cDNA synthesis was performed using second-strand synthesis reaction buffer, DNA polymerase I, and RNase H. Remaining overhangs were converted into blunt ends by exonuclease/polymerase activity. After adenylation of the 3′ ends of the DNA fragments, NEBNext adaptors with hairpin loop structures were ligated to the fragments to prepare them for hybridization. To select cDNA fragments that are 150–200 bp in length, the fragments in each of the library were purified with an AMPure XP system (Beckman Coulter, Brea, CA, USA). Then 3 μl USER Enzyme (NEB, Ipswich, MA, USA) was used with size-selected, adaptor-ligated cDNA at 37 °C for 15 min followed by 5 min at 95 °C before PCR. The qPCRs were performed with Phusion High-Fidelity DNA polymerase, Universal PCR primers, and Index (X) Primer. The PCR products were purified (AMPure XP system) and library quality was assessed on an Agilent Bioanalyzer 2100 system. Clustering of the index-coded samples was performed on a cBot Cluster Generation System using a TruSeq PE Cluster Kit v3-cBot-HS (Illumina, San Diego, CA, USA) according to the manufacturer’s instructions. After cluster generation, the paired-end sequencing (2*150 bp) were performed on an Illumina Hiseq2500 at the LC-BIO (Hangzhou, China).

### Reads mapping and transcriptome assembling

The 150 bp paired-end raw reads were firstly processed through FastQC to obtain the clean data, by removing the reads that contain sequencing adapter contaminations or poly-N and the low quality reads whose Q value were less than 20. At the same time, Q20, Q30 and GC content of the clean data were calculated. The clean reads from six cDNA libraries were merged and mapped to the *Gallus gallus* 5 (http://www.ensembl.org/Gallus_gallus/Info/Index) using TopHat and Bowtie v2.0.6 [28, 72]. The mapped reads of each sample were assembled using StringTie [29].Then, all transcriptomes were merged to reconstruct a comprehensive transcriptome using perl scripts. After the final transcriptome was generated, StringTie [29] and Ballgown [73] were used to estimate the expression levels of all transcripts. Specifically, ribosomal RNA in sequencing data was removed. We aligned all reads to ribosomal RNA of chicken download from ensemble by bowtie2, then those aligned reads were removed from fastq files.

### Coding potential and identification of lncRNAs

The known protein-coding transcripts and the transcripts whose length were smaller than 200 bp were firstly removed, and the remaining unknown transcripts were used to screen for putative lncRNAs. Then, the coding potential for the remaining transcripts was calculated by CNCI [74] and CPC [75]. A transcript was deemed to be lncRNA if the coding potentials were scored to be less than − 1 by CPC software and the coding potentials were scored to be less than 0 by CNCI software, which suggest that this transcript has no capacity of coding for proteins. Briefly, those candidate transcripts whose length were longer than 200 nt and the intersection between both coding prediction tools CPC and CNCI were deemed to be lncRNA.

### Analysis of differential expression patterns

Expression levels of all transcripts, including putative lncRNAs and mRNAs, were quantified as FPKM using the StringTie [29]. Based on Negative binomial distribution, differential gene expression was determined using DESeq with a *P* value < 0.05 and log_2_foldchange > 1 or < − 1 [76].

### GO and KEGG enrichment analysis of differentially expressed mRNA

Functional annotation of differentially expressed mRNA were performed based on Gene Ontology (GO) database and enriched pathways were analyzed using Kyoto Encyclopedia of Genes and Genomes database (KEGG).GO enrichment analysis for the screened differentially expressed mRNAs was carried out using GOseq platform [33]. The KEGG pathway enrichment analysis for the differentially expressed mRNAs was performed by using KOBAS software [34]. In these two analyses, *P* < 0.05 were defined as significantly enriched GO terms or KEGG pathways.

### Target gene prediction and functional enrichment analysis

To explore the function of lncRNAs, we predicted the target genes of lncRNAs in cis and trans. Cis-acting lncRNAs target neighboring genes [30]. We searched for coding genes 100-kb upstream and downstream of all the identified differentially expressed lncRNAs by using python script, according to the previously described method [77]. Some lncRNAs play trans-roles in regulating target genes through complementary base pairing (part region, default max trace back 50 nt). In the present study, based on the sequences of our identified differentially expressed mRNAs and lncRNAs, trans-regulated targets of the lncRNAs were identified according to the free energy (< − 11 kcal/mol) that needed to form the secondary structure using RIsearch [61]. Moreover, those lncRNAs and genes should house in different chromosomes. Briefly, the parameters of RIsearch were set as: “ext_len=50, ext_penalty=30, RIsearch_energy= -11”; and the linux shell command was ~/Softare/RIsearch1–1/RIsearch -q lncRNA.fasta -t mRNA.fasta -d 30 -l 50 > RIsearch_run_out.txt.

Then, we performed Gene Ontology (GO) and Kyoto Encyclopedia of Genes and Genomes (KEGG) analyses of the target genes for lncRNAs [76]. GO terms were enriched when *P* value was less than 0.05 using GOseq platform [33] and The KEGG pathways with a *P* < 0.05 were defined as significantly enriched pathways using KOBAS software [34].

### Real-time quantitative PCR

We selected 5 lncRNAs and 5 mRNAs represent different expression levels for further qRT-PCR analysis. Total RNA from 5 adult roosters’ and 20 one-day-old rooster’ livers were extracted using Trizol reagent (TaKaRa, Dalian, China). The RNA was quantified using a NanoDrop® ND-1000 spectrophotometer (Thermo Scientific, MA, USA) with the OD value set at 260 nm; the purity was assessed by determining the OD260/OD280 ratio and the quality (RNA degradation and contamination) was further assessed using formaldehyde-agarose gel electrophoresis. RNA samples from the 5 adult chickens and 20 one-day-old chicks were analyzed by qPCR.

About 1 μg of total RNA was reverse transcribed using the PrimeScript™ RT reagent Kit with gDNA eraser (TaKaRa, Dalian, China). qPCR were performed using SYBR® Green PCR Master Mix (TaKaRa, Dalian, China).A 25 μL PCR mixture was quickly prepared from 12.5 μL of SYBR® Premix ExTaq II (2×), 1 μL of forward primer (10 μM/L), 1 μL of reverse primer (10 μM/L), 1 μL of cDNA, and 9.5 μL of double-distilled water. Primers for β-actin (internal control genes) as well as the differentially expressed mRNAs and lncRNAs (Additional file 11) were designed using Primer-BLAST (https://www.ncbi.nlm.nih.gov/tools/primer-blast/index.cgi?LINK_LOC=BlastHome). The PCR with amplifications was conducted in an iCycler iQ5 multicolor real-time PCR detection system (Bio-Rad Laboratories) and programmed as follows: 95 °C for 10 min; 40 cycles of 95 °C for 10 s; 60 °C for 30 s; 72 °C for 30 s; and 72 °C for 5 min. All samples were examined in triplicate.

All data were analyzed using the 2^−ΔΔCt^ method [78]. The statistical evaluation of experimental results was analyzed by Student’s T test using SPSS 20.0 statistical software. All data were expressed as means with standard error (SE). Differences were considered to be statistically significant at *P* < 0.05.

## Additional files

Additional file 1: **Table S1.** Summary of protein-coding genes identified in the chicken liver libraries. (XLSX 4278 kb)

Additional file 2: **Table S2.** Summary of lncRNAs identified in the chicken liver libraries. 1. Class code: “x” represent the antisense lncRNAs, “o” represent the sense lncRNAs, “j” represent the intronic lncRNAs, “i” represent the intron lncRNAs, and “u” represent the intergenic lncRNAs; 2. Known/Novel: We used reference genome download on Ensembl (galgal 5). However, there weas few annotated lncRNA in gga5 gtf files. As a result, all novel assembled transcripts (> 200 bp) by cufflinks with non protein-coding potential were labelled as novel lncRNAs. (XLSX 290 kb)

Additional file 3: **Table S3.** Sequence information of all expressed lncRNAs found in the present study. (FA 69436 kb)

Additional file 4: **Table S4.** Summary of differentially expressed protein-coding genes between mature livers and immature livers in chickens. (XLSX 498 kb)

Additional file 5: **Table S5.** Summary of differentially expressed lncRNAs between mature livers and immature livers in chickens. (XLSX 32 kb)

Additional file 6: **Table S6.** GO enrichment analysis (*P* < 0.05) of the differentially expressed mRNAs. S gene number: the number of significant differentially expressed mRNAs which match to a GO term; TS gene number: the number of significant differentially expressed mRNAs which have GO annotations; B gene number: the number of detected mRNAs which match to a GO term; TB gene number: the number of all detected mRNAs which have GO annotations. (XLSX 39 kb)

Additional file 7: **Table S7.** The differentially expressed target protein-coding genes of the differentially expressed lncRNA in the present study. (XLSX 3850 kb)

Additional file 8: **Table S8.** GO enrichment analysis (P < 0.05) of differentially expressed protein-coding genes targeted by differentially expressed lncRNAs in either trans- or cis- regulatory roles. S gene number: the number of significant differentially expressed lncRNAs whose cis- or trans-regulated mRNAs match to a GO term; TS gene number: the number of significant differentially expressed mRNAs lncRNAs whose cis- or trans-regulated mRNAs have GO annotations; B gene number: the number of detected lncRNAs whose cis- or trans-regulated mRNAs match to a GO term; TB gene number: the number of all detected lncRNAs whose cis- or trans-regulated mRNAs have GO annotations. (XLSX 19 kb)

Additional file 9: **Table S9.** LncRNAs and its potential target genes that are involved in some key pathways related to the postnatal liver development. (XLSX 897 kb)

Additional file 10: **Table S10.** Quality parameters of the RNA samples used in Ribo-zero RNA Sequencing. (XLSX 9 kb)

Additional file 11: **Table S11.** Primers used in the qRT-PCR analysis. (XLSX 10 kb)
